# 1,4-Dithianes: attractive C2-building blocks for the synthesis of complex molecular architectures

**DOI:** 10.3762/bjoc.19.12

**Published:** 2023-02-02

**Authors:** Bram Ryckaert, Ellen Demeyere, Frederick Degroote, Hilde Janssens, Johan M Winne

**Affiliations:** 1 Department of Organic and Macromolecular Chemistry, Ghent University, Krijgslaan 281 (S4), 9000 Gent, Belgium

**Keywords:** 1,4-dithianes, 1,4-dithiins, 2,3-dihydro-1,4-dithiins, heterocycles, target synthesis

## Abstract

This review covers the synthetic applications of 1,4-dithianes, as well as derivatives thereof at various oxidation states. The selected examples show how the specific heterocyclic reactivity can be harnessed for the controlled synthesis of carbon–carbon bonds. The reactivity is compared to and put into context with more common synthetic building blocks, such as 1,3-dithianes and (hetero)aromatic building blocks. 1,4-Dithianes have as yet not been investigated to the same extent as their well-known 1,3-dithiane counterparts, but they do offer attractive transformations that can find good use in the assembly of a wide array of complex molecular architectures, ranging from lipids and carbohydrates to various carbocyclic scaffolds. This versatility arises from the possibility to chemoselectively cleave or reduce the sulfur-heterocycle to reveal a versatile C2-synthon.

## Introduction

**Scheme 1:**
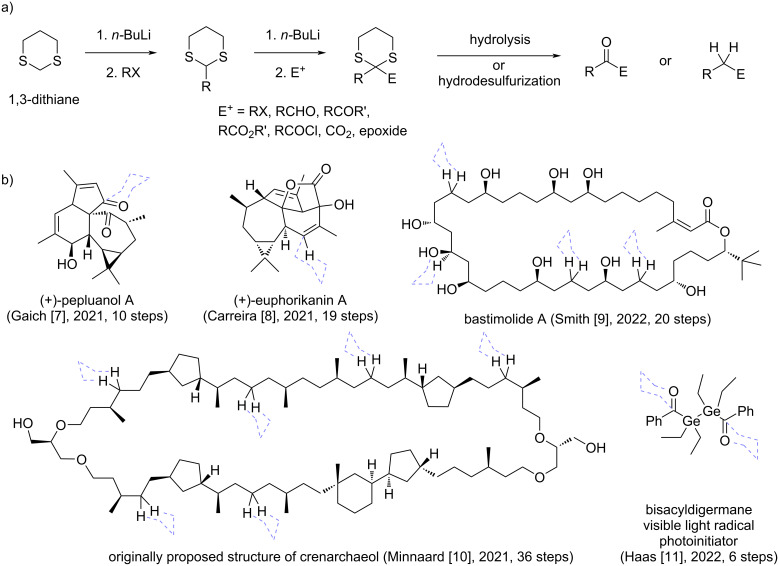
1,3-Dithianes as useful synthetic building blocks: a) general synthetic utility (in Corey–Seebach-type reactions) [1–6] and b) recent applications in the total synthesis of complex target products (original attachment place of 1,3-dithiane ‘scaffolding’ groups shown with dashed purple lines) [7–11].

1,3-Dithianes are text book examples of versatile organic synthesis building blocks. They are familiar carbonyl protecting groups, but are more commonly known as ‘umpolung’ reagents, or acyl anion equivalents [1–6]. This is because they can be readily metalated and alkylated, allowing the rapid build-up of target molecules (see Scheme 1a) [7]. Once the important skeletal carbon–carbon bonds have been formed around the thioketal carbon, the sulfur-heterocycle can perform its primary function as a temporary protecting group and be chemoselectively hydrolyzed to afford a carbonyl functional group. Alternatively, the carbon–sulfur bonds can be chemoselectively hydrogenolyzed to reveal a methylene moiety. Since their introduction into synthesis by the work of Corey and Seebach [1], the use of 1,3-dithianes as powerful C1-synthons has significantly expanded from this original strategic scheme, and several excellent reviews have highlighted their synthetic versatility and utility in the assembly of complex target molecules [4–6]. Some recent examples of 1,3-dithiane-mediated short and efficient total syntheses of complex target products are shown in Scheme 1b [7], wherein the original ‘dithiane-scaffolding’ is indicated on the structures of the final targets.

**Scheme 2:**
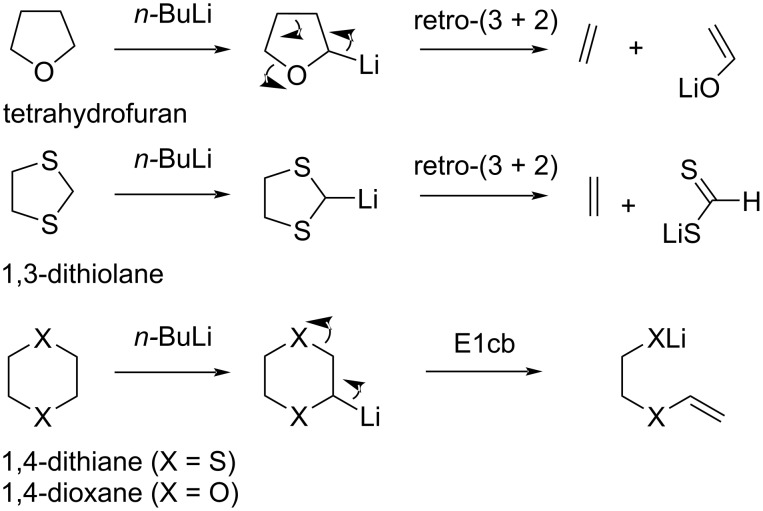
Metalation of other saturated heterocycles is often problematic due to β-elimination [16–17].

Compared to the very accomplished 1,3-dithianes, not many other sulfur-heterocycles have been able to follow into the mainstream organic synthesis tool box. For example, 1,3-dithiolanes are underperforming as heterocyclic building blocks with respect to their homologous counterparts. This is mainly due to their problematic metalation reactions (Scheme 2). Many ‘olane-type’ saturated heterocycles do not afford stable metalated species [12–15]. The most well-known example of this is tetrahydrofuran, which decomposes in a reverse (3 + 2) cycloaddition which expels a heteroatomic anion via a β-elimination-type mechanism somewhat similar to a classical Grob-type fragmentation. Similarly, 1,3-dithiolanes undergo fragmentation at relatively low reaction temperatures, limiting their synthetic application to alkylation with highly reactive electrophiles. The homologous 1,4-dithianes or 1,4-dioxanes are readily available as simple building blocks, but also undergo a swift β-elimination following the metalation of one of their ring carbons, again limiting their appeal as building blocks [16–17].

**Scheme 3:**
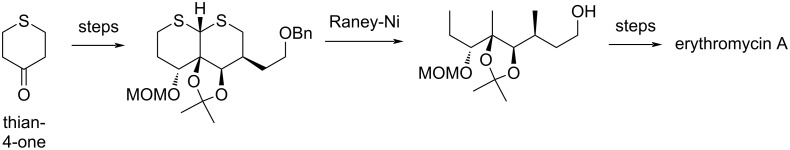
Thianes as synthetic building blocks in the construction of complex molecules [18].

In his landmark synthesis of erythromycin A [18], Woodward famously introduced thianes and ring-fused thianes (dithiodecalins) as building blocks for polyketide chain fragments, in order to be able to exploit their cyclohexane-like conformational behavior in the control of the relative stereochemistry along the polyketide backbone (Scheme 3). The synthetic implementation of thianes, however, is not straightforward, as their elaboration through – for example – tethered aldol-type reactions requires many steps. Moreover, thianes are also quite particular heterocyclic building blocks as C5-synthons for linear chain fragments, and lack generality in this aspect.

**Figure 1:**
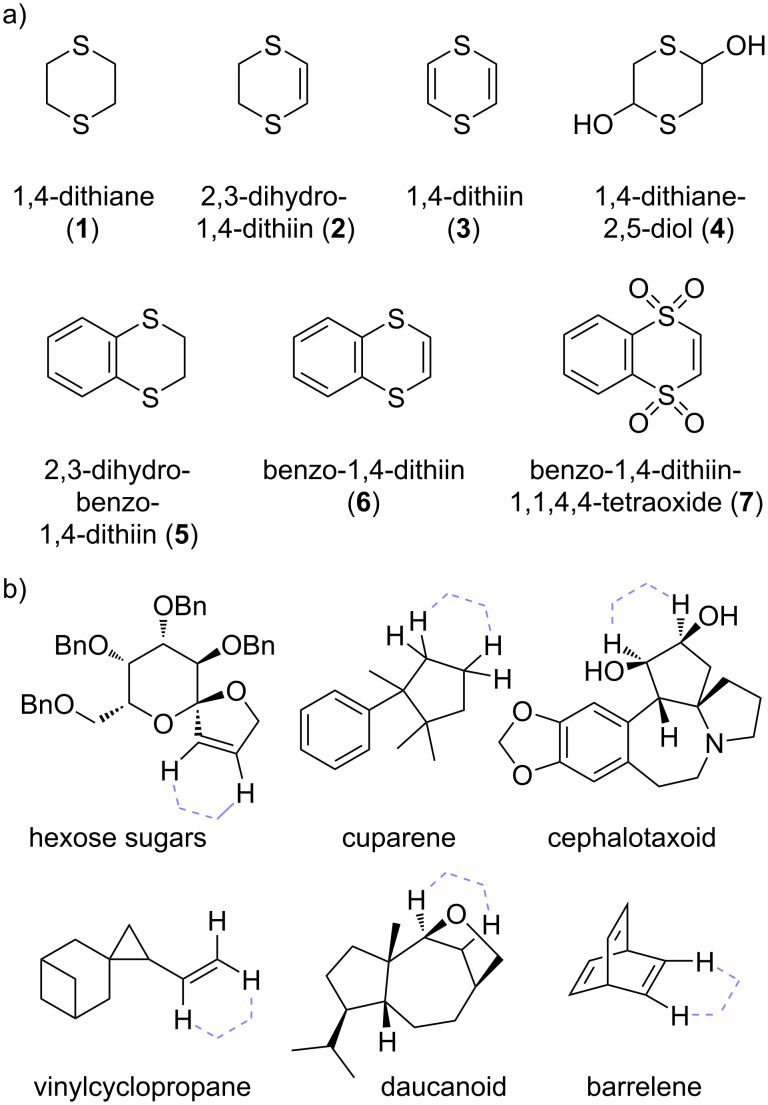
a) 1,4-Dithiane-type building blocks that can serve as C2-synthons and b) examples of complex target structures that have been prepared using 1,4-dithiane-type building blocks (see review, original attachment place of 1,4-dithiane ‘scaffolding’ groups shown with dashed purple lines), for references, see text of following chapters.

In principle, 1,4-dithianes should offer good options for the development as C2-synthons that are complementary in scope to the more widely used 1,3-dithianes, but the β-fragmentation problem (cf. Scheme 2) hampers their easy derivatization. It should be noted that this elimination problem can be partially circumvented by employing the related unsaturated 1,4-ditthiin or 1,4-dihydrodithiin compounds, and that more options for bond formation are available in oxidized derivatives (vide infra). In this review, we will focus on developments in the application of 1,4-dithiane building blocks, including their unsaturated and oxidized derivatives (Figure 1a). The chemistry of fully unsaturated 1,4-dithiins have received a good deal of attention in synthesis [19–23], in particular as these heterocycles constitute non-aromatic (and non-planar) analogues of thiophenes that find use in materials science applications. The properties, synthesis and materials applications of 1,4-dithiins have recently been reviewed by Etkind and Swager [24], and will not be extensively covered in this review. Instead, the current review will focus on 1,4-dithiin and 1,4-dithiane building blocks used in the synthesis of complex target molecules, wherein one of the ethylenedithio fragments can be regarded as a temporary, strategic tethering group that facilitates bond-formation reactions, just as in classical Corey–Seebach-type reactions. The literature examples that fit to this concept, developed by our own research group as well as others, are so far still limited, but they are at the same time quite diverse, as can be seen in the selected examples of target compounds shown in Figure 1b.

## Review

### 1 Availability and synthesis of 1,4-dithiane- and 1,4-dithiin-type building blocks

**Scheme 4:**
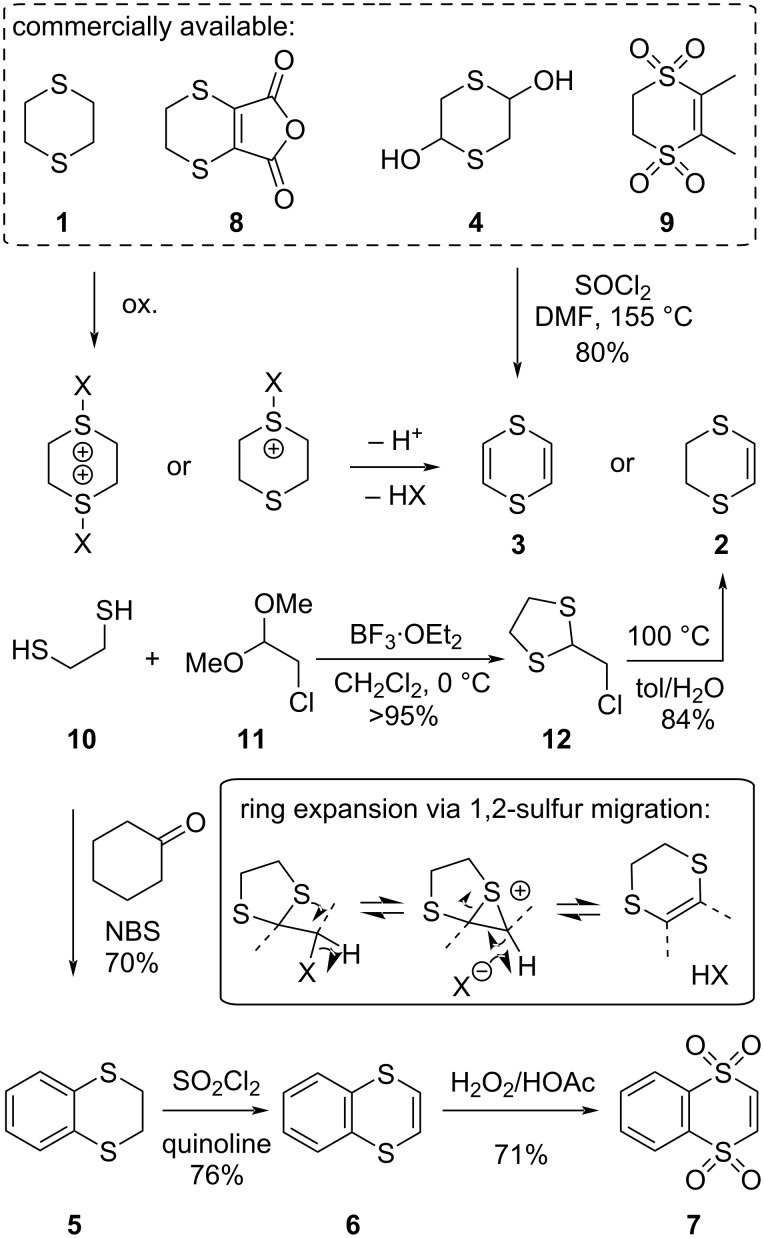
Synthetic availability of 1,4-dithiane-type building blocks.

While the synthesis of 1,4-dithiane-type ring systems is not the primary focus of this review, the synthetic accessibility of these building blocks is of course an important consideration, and it impacts the utility of any building block-based strategy [19–24]. The commercially available 1,4-dithiane derivatives are relatively limited (see Scheme 4, top frame), but many of these heterocycles can be easily prepared from simple and widely available organic building blocks.

The parent fully saturated 1,4-dithiane (**1**) is commercially available, but has limited synthetic application, as the functionalization of this heterocycle is quite challenging due to the ease of the β-fragmentation pathway of lithiated derivatives (Scheme 2). Chlorination or oxygenation of the ring sulfur atom(s) in **1**, followed by Pummerer-type rearrangement and elimination, affords a straightforward access to the more useful unsaturated versions 1,4-dithiin (**3**) and 5,6-dihydro-1,4-dithiin (**2**, Scheme 4) [25]. However, more convenient synthetic procedures exist for the synthesis of these two basic heterocycles.

For the synthesis of large amounts of 1,4-dithiin (**3**), this can be most conveniently achieved by a double dehydration of the commercially available 1,4-dithiane-2,5-diol (**4**) [26]. The diol **4** is commercially available in large quantities and is formally also a 1,4-dithiane derivative, but except for its dehydration to 1,4-dithiin, it is almost exclusively used as a source of mercapto acetaldehyde (of which it is a direct cyclic hemithioacetal dimer). The chemistry and synthetic applications of reagent **4** and related α-thiocarbonyl substances have been reviewed in detail elsewhere [27], and they are rarely used as building blocks for 1,4-dithiane-type targets.

For the synthesis of 5,6-dihydro-1,4-dithiin (**2**), several alternative approaches exist. The direct dehydrogenation of 1,4-dithiane is somewhat cumbersome [25], and, moreover, 1,4-dithiane (**1**) is surprisingly not available in large quantities at a reasonable cost, in spite of its apparent simplicity. Our lab has found that the synthetic method for dihydrodithiins pioneered by Parham and co-workers to be the most suited (Scheme 4, **12** → **2**) [28–30]. Parham described a ring expansion of 1,3-dithiolanes derived from α-halocarbonyls into the 1,4-dithianes which then dehydrohalogenate to afford dihydrodithiins. The ring expansion involves a 1,2-sulfur migration of a β-haloalkylsulfide via a cyclic sulfonium intermediate, which then ring opens and eliminates the halide to give the unsaturated 1,4-dithiane ring. We have found this Parham ring expansion to be the most practical preparatory procedure for **2** on large scale [30]. Thus, a simple condensation of ethane-1,2-dithiol (**10**) and the dimethylacetal derived from chloroacetaldehyde **11** affords the dithiolane **12**. This 1,3-dithiolane spontaneously rearranges to the 1,4-dithiane with an elimination of hydrochloric acid by refluxing in a mixture of water and toluene. This two-step procedure constitutes a scalable and simple access to building block **2**.

The benzannelated series of 1,4-dithiane heterocycles **5**–**7** can in principle be obtained using Parham’s α-halocarbonyl condensation and rearrangement approach, starting from benzene-1,2-dithiol. More conveniently, however, ethanedithiol and cyclohexanone can be condensed, and then brominated with an excess of bromination reagent, which effects the 1,2-sulfur-migratory ring expansion, followed by bromination-induced dehydrogenation to the aromatic ring [31]. At the time of writing this review, the resulting benzoannelated dithiane **5** is also commercially available in small quantities from a limited number of suppliers. Oxidation and dehydration of **5** to the fully unsaturated ring system **6** is quite straightforward (especially compared to the same reactions on 1,4-dithiane (**1**)), and yields the useful building block **6**.

For the availability of the disulfone series of derivatives of 1,4-dithianes and 1,4-dithiins (1,4-dithiin-1,1,4,4-tetraoxides), it should be noted that the dimethyl derivative **9** has actually been commercialized as the defoliating herbicide dimethipin [32]. Other 1,4-dithiin tetraoxides can be easily derived from the corresponding sulfides by treatment with an excess of a perbenzoic acid, as shown for the oxidation of **6** to **7** [33–34]. Partial oxidations are also possible, but lead to mixtures of sulfoxides, including *cis*- and *trans*-sulfoxide stereoisomers (see also chapter 6).

For a more detailed and extensive discussion of the synthesis of 1,4-dithiin derivatives, and the various strategies that are available, we refer to other reviews in this area [19–24]. In our hands, the ring expansion of 1,3-dithiolanes as pioneered by Parham is the most generally applicable and most versatile strategy, especially for substituted 1,4-dithiane ring systems, as a wide range of α-halocarbonyl starting materials can be used in this scheme.

### 2 Reactivity of 1,4-dithiins as pseudo-aryl substrates

**Scheme 5:**
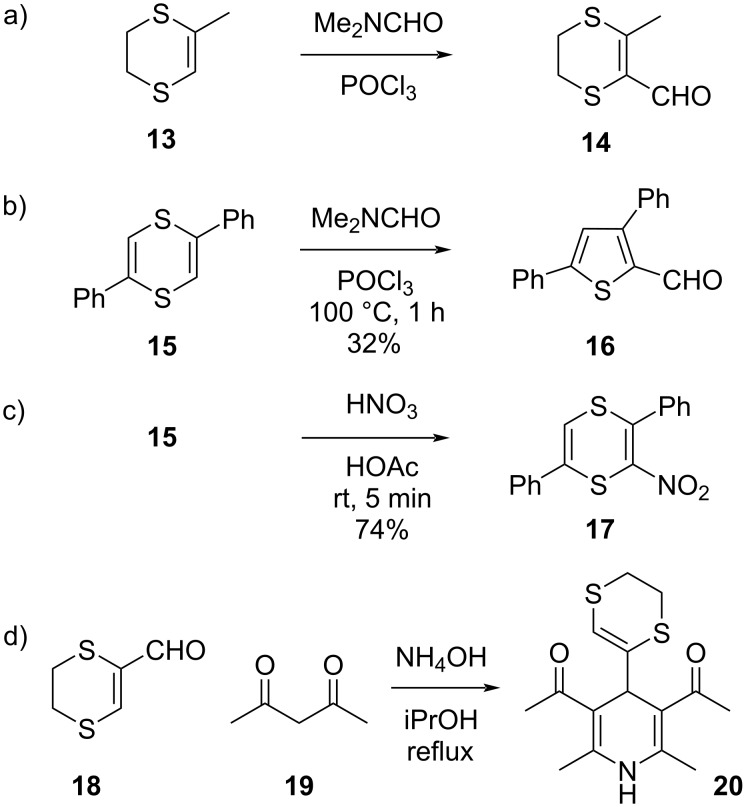
Dithiins and dihydrodithiins as pseudoaryl groups [36–39].

Based on simple Hückel considerations for cyclic unsaturated hydrocarbons, 1,4-dithiin (**3**) could be considered as an antiaromatic compound [24]. For heterocycles, antiaromaticity is usually not a very relevant concept, as there will be no degeneration of the molecular orbitals. Nevertheless, 1,4-dithiin does adopt a non-planar boat-like conformation, which can rapidly interconvert through a planar, but nonaromatic geometry (with a shallow folding energy curve of less than 12 kJ/mol) [35]. Because of the extensive conjugation between two sulfurs and the vinyl bond, the resulting six-electron system (S–C=C–S) can be regarded as a pseudoaromatic core that tends to conserve itself in organic reactions just like an aromatic sextet would. Indeed, some classical transformations of aryl substrates have also been reported for thiovinyl ethers, and also for dihydrodithiins (Scheme 5), although there are obvious limitations to this point of view.

Classical electrophilic aromatic substitution procedures such as the Vilsmeier–Haack reaction or a simple nitration have been reported for vinyl sulfides, including dihydrodithiin **13** (Scheme 5a) [36]. In fact, Parham has found that fully unsaturated dithiins can undergo this electrophilic formylation, but at the same time also undergo a ring contraction and an aromatizing desulfurization to yield thiophenes as the main formylated products (viz **16**, Scheme 5b) [37]. Electrophilic aromatic substitution under less forcing reaction conditions of the same substrate **15**, using a room temperature nitration procedure, does yield the expected mononitrated dithiin **17** in good yield, without desulfurization [38].

1,4-Dithiin-2-carbaldehyde (**18**) may superficially look like a sulfur-substituted acrolein derivative, with a reactive vinylogous thioester moiety, but in fact their reactivity is more akin to that of an arylaldehyde, as shown by the example of the synthesis of Hantzsch ester **20** from a condensation of ammonia with **18** and acetylacetone (**19**, Scheme 5c) [39].

**Scheme 6:**
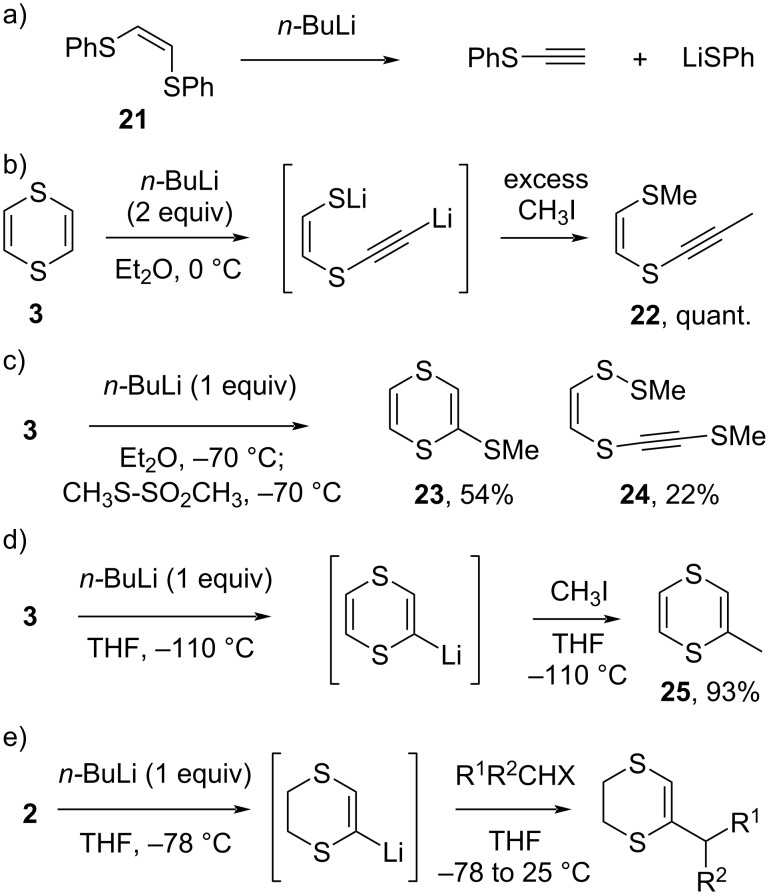
Metalation of other saturated heterocycles is often problematic due to β-elimination [40–42].

As mentioned in the Introduction, the metalation of 1,4-dithianes is quite problematic [12,16]. In the unsaturated 1,4-dithiin series, however, the metalation of the cyclic vinyl-1,2-disulfide moiety is somewhat more feasible. Even though linear vinyl-1,2-disulfides such as **21** cannot be lithiated to give a stable vinyllithium species, and immediately expel a sulfide anion to afford phenylthioacetylene (Scheme 6a) [40], Brandsma showed that the lithiated derivatives of 1,4-dithiin (**3**) can be generated by ‘*ortho*-lithiation’-type reactions at −110 °C in THF (Scheme 6d) [41]. At 0 °C, attempts to generate the ‘*ortho*-lithiated’ dithiins resulted in quantitative ring opening, giving the dimethylated derivative **22** as the sole product (Scheme 6b). The lithiated 1,4-dithiins are also not stable at −70 °C in diethyl ether, and cannot be alkylated using methyl iodide. With a more reactive sulfanylation reagent, the intermediate lithiated species can be captured (Scheme 6c), and sulfanylated derivative **23** is isolated as the major product. However, the sulfanylated alkyne **24** is also already prominent at −70 °C, even though the metalation was not yet complete (as judged by recovered starting material **3**). These results indicate that the metalation is slow at −70 °C, and that at the same time already significant β-elimination is happening at this low temperature. In the better coordinating solvent THF, metalation is much more swift, even at −110 °C (Scheme 6d), and the generated lithiated dithiin intermediate can be almost quantitatively alkylated with iodomethane, giving dithiin **25**.

**Figure 2:**
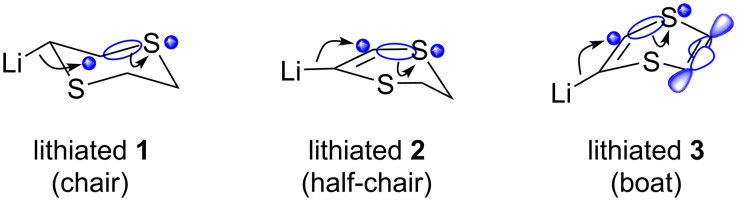
Reactive conformations leading to β-fragmentation for lithiated 1,4-dithianes and 1,4-dithiin.

In later studies, Palumbo and co-workers showed that the lithiated derivatives of 5,6-dihydro-1,4-dithiin (**2**) can survive until even higher temperatures (above −70 °C) before showing the expected fragmentation (Scheme 6e) [42]. The metalated dihydrodithiins can thus also be reacted with less activated electrophiles, including secondary alkylating agents. As we shall discuss in chapter 4, this makes 5,6-dihydro-1,4-dithiins the most suitable building blocks for Corey–Seebach-type alkylations. Herein, the lithiated sulfur-heterocycles act as a *cis-*vinyl anion equivalent, a strategy that was developed by Palumbo and co-workers. The method shows some complementarity to the more classical acetylene alkylations, followed by partial hydrogenation to the *cis*-olefin (see Scheme 10 and Scheme 11). The fact that lithiated **2** is more stable than its more unsaturated counterpart derived from dithiin **3**, can possibly be related to stereoelectronic ground state effects in **3** (Figure 2), wherein the non-planar geometry of the nonaromatic ring system can already line up the breaking (C–S) bond with the π-type orbitals on the unsaturated carbons on the eliminated alkenylsulfide chain fragment, leading to resonance stabilization. This effect is absent in lithiated **2**. For the fully saturated lithiated 1,4-dithiane derived from **1**, the higher intrinsic reactivity of the tetragonal alkyllithium species can explain the faster ring fragmentation pathway.

**Scheme 7:**
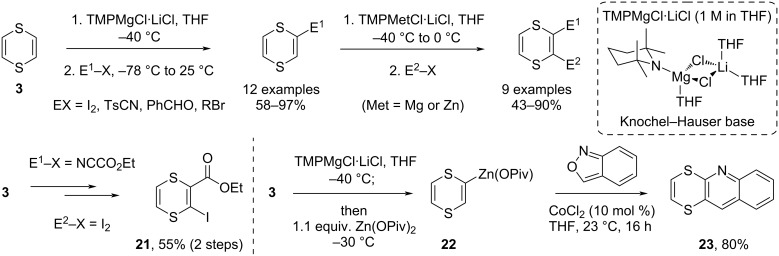
Mild metalation of 1,4-dithiins affords stable heteroaryl-magnesium and heteroaryl-zinc-like reagents that can be used in coupling reactions at higher temperatures [43–44].

More recently, Knochel and co-workers developed a higher temperature metalation reaction for 1,4-dithiins, using turbo-Hauser-type bases (Scheme 7) [43]. This allows the selective magnesiation or zincation of 1,4-dithiin (**3**), respectively, at −40 °C and 0 °C, temperatures at which these organometallic reagents are also reported to be quite stable. The zincated dithiins can also be prepared by transmetalation of the magnesiated dithiins at −30 °C, and these organozinc reagents can then be used in room temperature Pd-catalyzed cross-coupling reactions, as pseudo-heteroarylzinc reagents. Another example developed by Knochel uses the zincated 1,4-dithiin **22** as a nucleophile to add across the N–O bond in anthranil [44], which spontaneously cyclizes to a heterocycle-fused quinoline via a Friedel–Crafts-type pathway onto the released aldehyde moiety, a reactivity mode normally observed for electron-rich heteroarylzinc species. This mild metalation reaction of dithiins, and the unique stability of the organozinc derivatives further opens the door for synthetic applications of these heterocycles that aim to conserve the dithiin ring system, including cross-coupling-type chemistries on a conformationally stable *cis*-vinyl zinc building block.

### 3 Diels–Alder reactivity of 1,4-dithiin-based dienophiles and dienes

**Scheme 8:**
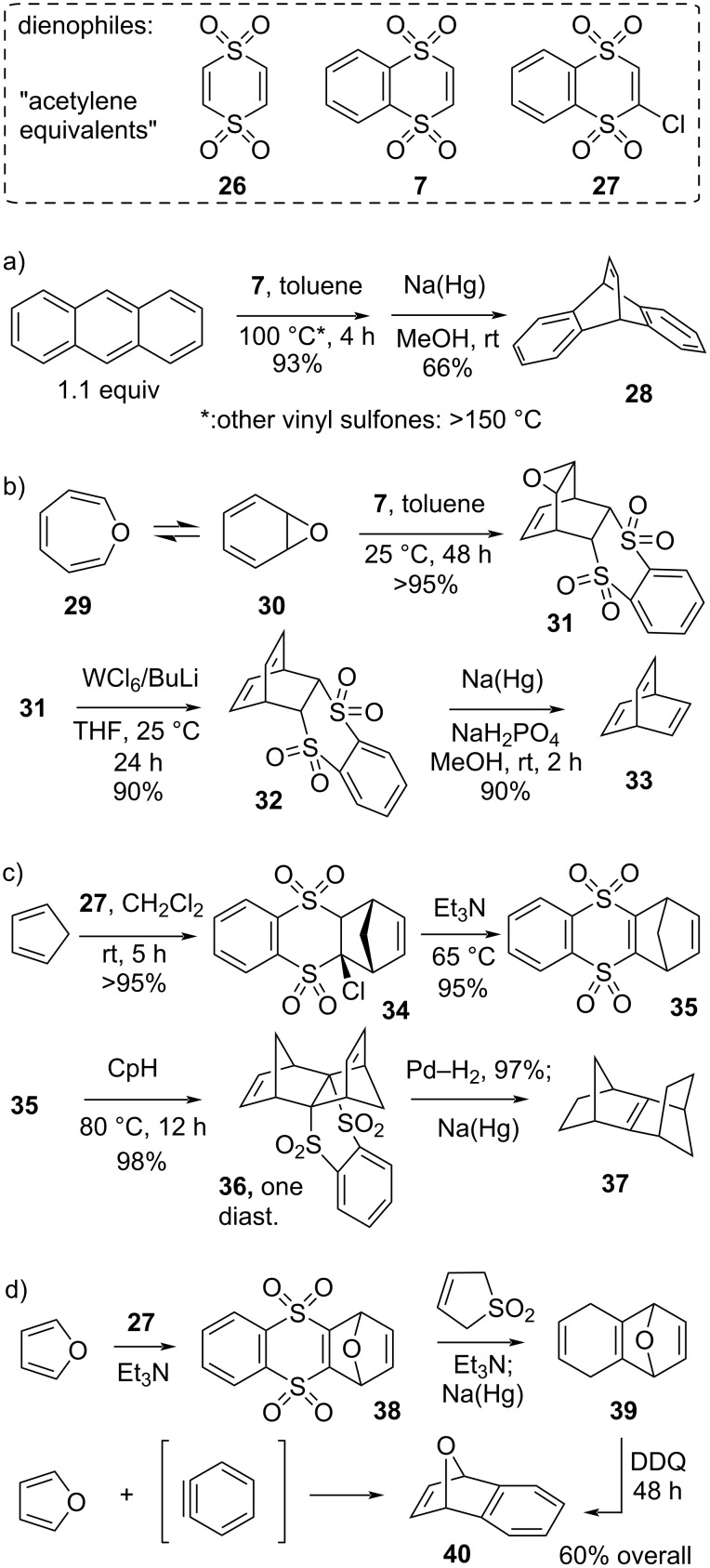
Dithiin-based dienophiles and their use in synthesis [33,49–54].

Vinyl sulfones and vinyl sulfoxides are classical synthetic equivalents of ethylene in Diels–Alder reactions, and have been widely used in total syntheses [45–48]. However, while vinyl sulfones are considerably more reactive than unactivated C2-dienophiles, they still show limitations. A series of vinyl-1,2-disulfone derivatives has been developed and investigated by De Lucchi for their use as synthetic equivalents of acetylenes in Diels–Alder reactions [49], since desulfonylation of the cyclohexene-disulfones with sodium amalgam or samarium(II) iodide affords 1,4-cyclohexadienes (Scheme 8). In a comparative study by Nakayama [50], the dithiin-type cyclic disulfone **7** emerged as a much stronger dienophile than any other simple linear vinyl disulfone (Scheme 8a), and by intermolecular competition experiments it was shown to be about as reactive as maleic anhydride. Benzo-1,4-dithiin-1,1,4,4-tetraoxide (**7**) also serves as an excellent acetylene equivalent through desulfonylation with sodium amalgam. The non-benzo-fused analog **26** should also be a reactive dienophile [51], but is a less useful building block, as it reacts twice and the adducts will not be as easily desulfonylated.

The dienophile **7** reacts with a wide range of dienes at room temperature, without the need for a Lewis acid catalyst. This is particularly helpful when dealing with sensitive dienes. A nice illustration of this is afforded by De Lucchi’s simple synthesis of barrelene (**33**) from oxepin (**29**, Scheme 8b) [52]. Oxepin’s equally unstable valence tautomer **30** (benzene oxide) is quite reactive as a diene in Diels–Alder reactions, and can react with **7** at room temperature to form the expected Diels–Alder adduct **32**, while non-cyclic vinyl disulfones require heating to 80 °C for 20–48 h. Deoxygenation of the epoxide and desulfonylation with sodium amalgam affords barrelene (**33**) in an excellent overall yield from oxepin.

The chlorinated 1,4-dithiin-derived dienophile **27** can be used as a ‘linchpin’ reagent (Scheme 8c), as a first Diels–Alder reaction can be followed by a straightforward base-promoted β-elimination of the chloride, releasing another reactive dienophile. This way, highly congested ring systems can be built up around the dithiin ring system, such as the *C*_2_-symmetrical sesquinorbornene **37** [33,53], but also asymmetrical congested ring systems can be accessed using this strategy. Finally, De Lucchi also designed a high yielding multistep sequence in which **27** can be used as a bench-stable alternative reagent for benzyne (Scheme 8d) [54]. For example, after a first Diels–Alder reaction with furan followed by dehydrochlorination, the resulting dithiin-tetroxide dienophile **38** is reacted with sulfolane (as a buta-1,3-diene precursor), to elaborate a propellane system with a fused cyclohexene ring. Reductive desulfonylation of the dithiane-tetroxide ring gives the cyclohexa-1,4-diene intermediate **39**. This intermediate can then be easily oxidized to afford the aromatic adduct **40**, which is the known cycloaddition product of furan and benzyne. Although this synthetic equivalent of benzyne requires a lengthy work-around, all synthetic operations are straightforward and high yielding.

**Scheme 9:**
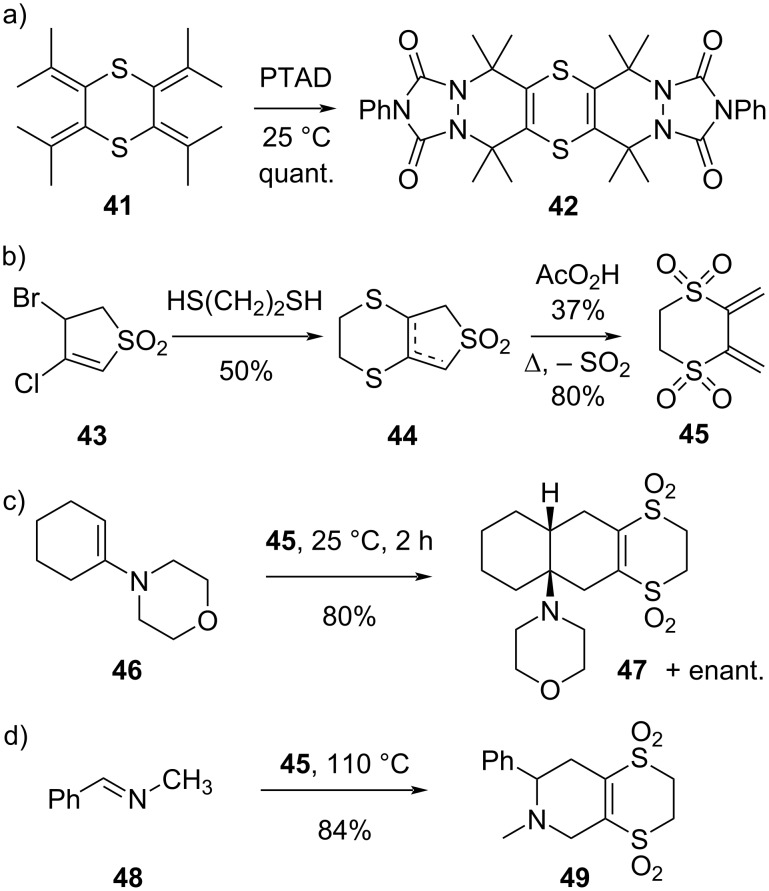
Dithiin-based dienes and their use in synthesis [55–57].

The concept of tethering Diels–Alder reaction partners with a temporary 1,4-dithiane-type ring has also been successfully extended to dienes (Scheme 9). Here, its strategic value is very straightforward, as it can be used to lock linear dienes into their reactive single-*cis* conformation. Ando and co-workers prepared the symmetrical 1,4-dithiane **41** (Scheme 9a) [55], and amply illustrated the concept by reacting it in a [4 + 2] cycloaddition with a highly reactive diazo dienophile (Cookson’s reagent or 4-phenyl-1,2,4-triazoline-3,5-dione (PTAD)) to afford the bis-adduct dithiin **42** in quantitative yield. Although 2,5-dimethylhexa-2,4-diene cannot possibly adopt a coplanar single*-cis* conformation, and is therefore completely unreactive in Diels–Alder reactions as a diene, its 1,4-dithiane-tethered version in **41** must show a sufficient proximity between the terminal carbons of the diene to allow a Diels–Alder-type reaction.

Chou and co-workers developed an elegant synthesis of a 1,4-dithiane-fused sulfolane **45** (Scheme 9b) [56]. Oxidation of the 1,4-dithiane-fused sulfolane **44** to the hexoxide and heating to 130 °C afforded the pure 1,4-dithiane-tethered diene **45** via the usual retro-chelotropic reaction of sulfolanes. Attempts to generate the corresponding simple 1,4-dithiane-fused butadiene in the same way directly from sulfolane **44** actually failed to give the expected 1,4-dithian-tethered butadiene derivative, which was so unstable that it resisted isolation. Thus, only the diene-disulfone reagent **45** has been investigated for its Diels–Alder reactivity. As expected by the strong electron-withdrawing nature of the two sulfones, the diene **45** showed quite poor reactivity towards classical Diels–Alder dienophiles, affording the adducts in only 10–20% yield (results not shown in scheme). Conversely, in reactions with electron-rich olefins, better yields are observed for the inverse electron demand Diels–Alder reaction (Scheme 9c). For example, the enamine **46**, derived from cyclohexanone and morpholine, was readily annulated by **45** to afford the decalin ring system **47** in good yield. Similarly, simple imines like **48** were found to be rather effective aza-dienophiles for this electron-poor single*-cis*-locked diene (Scheme 9d). Somewhat unexpectedly, and in contrast to the results from Nakayama [50] and De Lucchi [33,53–54], the dithiin-type vinyl disulfones like **47** and **49** actually resisted all attempts to further undergo a Diels–Alder reaction with normal dienes, even for highly reactive ones like cyclopentadiene. As a conclusion, the additional benzo-ring fusion seems to be an important factor in the swift propellane-forming reactivity of dithiin-type dienophiles **7** and **27**.

### 4 Alkylation chemistry of 1,4-dithianes and 1,4-dithiins: stereocontrolled synthesis of *Z*-alkenes

**Scheme 10:**
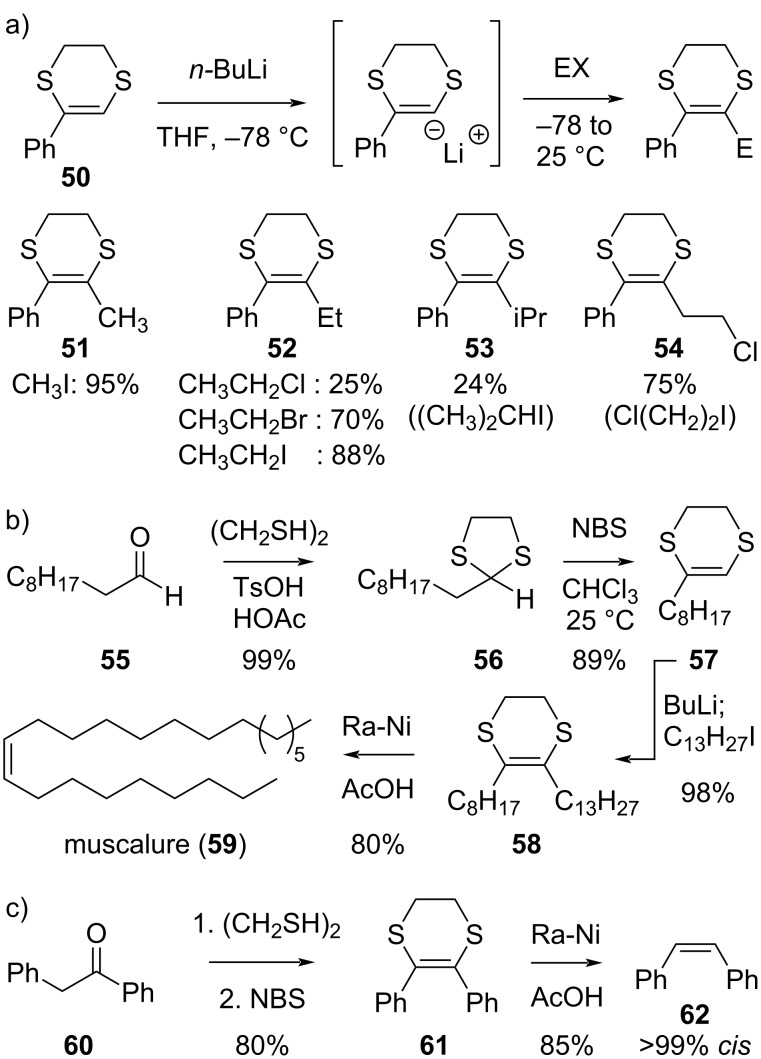
Stereoselective 5,6-dihydro-1,4-dithiin-based synthesis of *cis*-olefins [42,58].

As mentioned in chapter 2 of this review, Palumbo and co-workers found that 5,6-dihydro-1,4-dithiins are relatively resistant to β-elimination in their lithiated form (Scheme 6e), and this opens up options for Corey–Seebach-type alkylation reactivity with a wider range of electrophiles. The method is demonstrated by the smooth lithiation and subsequent alkylation of the acetophenone-derived dithiin **50** (Scheme 10a) [42]. Palumbo’s elegant overall approach to dihydrodithiin-mediated synthesis starts from a carbonyl compound, wherein an aldehyde can undergo ‘umpolung’ into a *cis*-vinyl anion equivalent via a 1,3-dithiolane-to-1,4-dithiane rearrangement (Scheme 10b). The potential of the method is demonstrated by the synthesis of (*Z*)-9-tricosene or muscalure (**59**), which is the natural sex pheromone of the common house fly [58]. The aldehyde **55** is converted into a vinyl anion equivalent **57** in two high yielding and operationally simple steps. The alkylation of this dihydrodithiin intermediate proceeds extremely smoothly, yielding the 1,4-dithiane-tethered version of the target molecule (**58**). A chemoselective and stereospecific mild hydrodesulfurization with Raney nickel in acetic acid affords the target compound **59** as a single *Z*-diastereomer. Interestingly, glacial acetic acid proved to be the only solvent that could avoid both undesired overreduction and *cis*–*trans* isomerization of the alkene bond. It should be noted that the three-step strategy (dithiolane protection, oxidative rearrangement, and hydrodesulfurization) can be used to treat carbonyl functional groups as synthetic equivalents of *cis*-alkenes (Scheme 10c), as is shown by the highly stereoselective conversion of α-phenylacetophenone (**60**) into *cis*-stilbene (**62**), via the dihydrodithiin intermediate **61** [58].

**Scheme 11:**
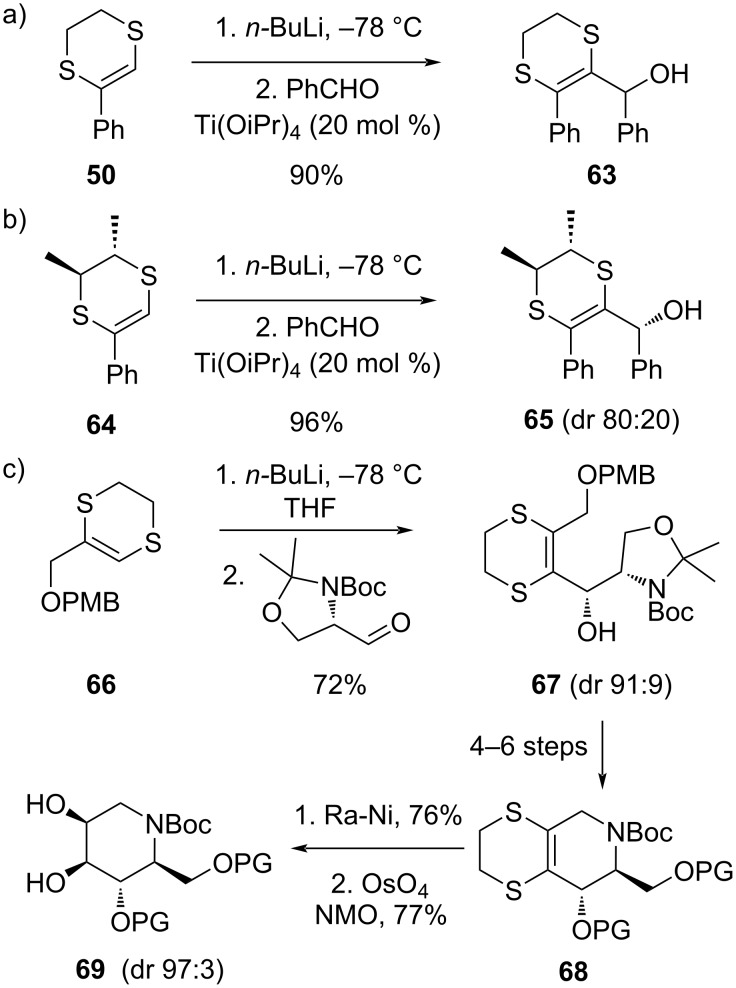
Addition to aldehydes and applications in stereoselective synthesis.

The addition of lithiated dihydrodithiins with aldehydes, epoxides or ketones are also all feasible, but sometimes require some more attention (Scheme 11). Palumbo and co-workers have found that substituted dithiins such as **50** can give difficulties (Scheme 11a) [42]. The reactivity of the oxy-electrophiles can be enhanced by adding a Lewis acid catalyst such as titanium(IV) isopropoxide [59]. In this way, also epoxides can be smoothly reacted with lithiated dithiins, and both allyl and homoallyl alcohols can thus be prepared in a stereocontrolled manner. Stereogenic centers on the ethylenedithiol tethering group can give modest levels of stereoinduction (Scheme 11b), despite the relatively remote relationship between the stereogenic centers [60–61]. Guaragna, Palumbo, D’Alonzo and co-workers have very productively used this approach in a number of elegant stereocontrolled syntheses of polyhydroxy compounds and (derivatives of) natural carbohydrates, centering around Palumbo’s versatile building block **66** (Scheme 11c) [62–80]. The PMB-protected allyl alcohol **66** can be lithitated and reacted with a range of electrophiles, even without the need for a Lewis acid catalyst, and good levels of stereoinduction can be achieved. The method was used for the synthesis of a range of hexose sugars, as well as iminosugars (viz **66** → **67** → **68**), wherein the piperidine-fused dihydrodithiin ring is first hydrodesulfurized and then dehydroxylated in a stereospecific and stereoselective manner [62]. The dihydrodithiin building block **66** thus acts as a synthetic equivalent of an allyl alcohol anion and serves as a versatile synthon for de novo carbohydrate synthesis [63].

**Figure 3:**
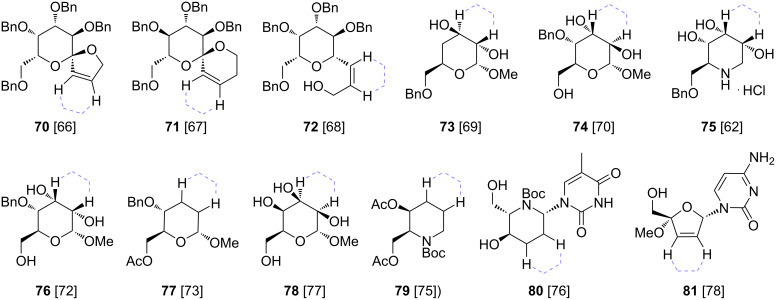
Applications in the total synthesis of complex target products with original attachment place of 1,4-dithiane ‘scaffolding’ groups shown with dashed purple lines, references indicated with structures.

The alkylation of dihydrodithiins via lithiation and reaction with electrophiles is quite versatile, as shown by the examples above, but is still limited in scope, especially compared to 1,3-dithianes. This is mainly due to the still limited stability of the lithiated species (vide supra), limiting its use mainly to reactions with more reactive electrophiles, and often leading to incomplete reactions or the need to precisely control the reaction conditions. Nevertheless, the scope established so far is quite wide as can be seen in Figure 3 for some representative examples of targets, wherein the used dihydrodithiin-scaffolding is tentatively indicated on the target structures **70**–**81** [62,64–80]. D’Alonzo, Palumbo, Guaranga and co-workers also developed a de novo synthesis of the unnatural enantiomer of the iminosugar drug miglustat [81]. Miglustat is a biologically active analog of the natural product deoxynojirimycin, and its enantiomer also shows a promising profile in early biological activity studies.

**Scheme 12:**
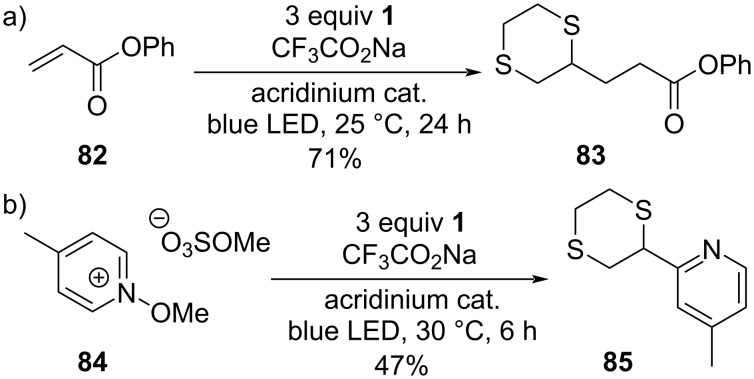
Direct C–H functionalization methods for 1,4-dithianes [82–83].

In future applications of 1,4-dithiane or -dithiin building blocks, the recently described zincation protocol by Knochel and co-workers could offer more opportunities here (Scheme 7) [43–44]. Furthermore, some recently reported photoredox-catalyzed thioether (C–H) alkylation and heteroarylation reactions developed by Alfonzo and Hande have demonstrated the use of 1,4-dithianes as viable substrates in such attractive bond-formation processes (Scheme 12) [82–83]. These recently obtained results indicate that a sulfur-stabilized carbon radical derived from 1,4-dithiane (**1**) is a viable reaction intermediate, opening up the use of 1,4-dithianes in various free radical-type cross-couplings.

### 5 Synthetic equivalents of the allyl cation in (3 + 2) cycloadditions: 5,6-dihydro-1,4-dithiin-2-methanol as a stabilized allyl cation

**Scheme 13:**
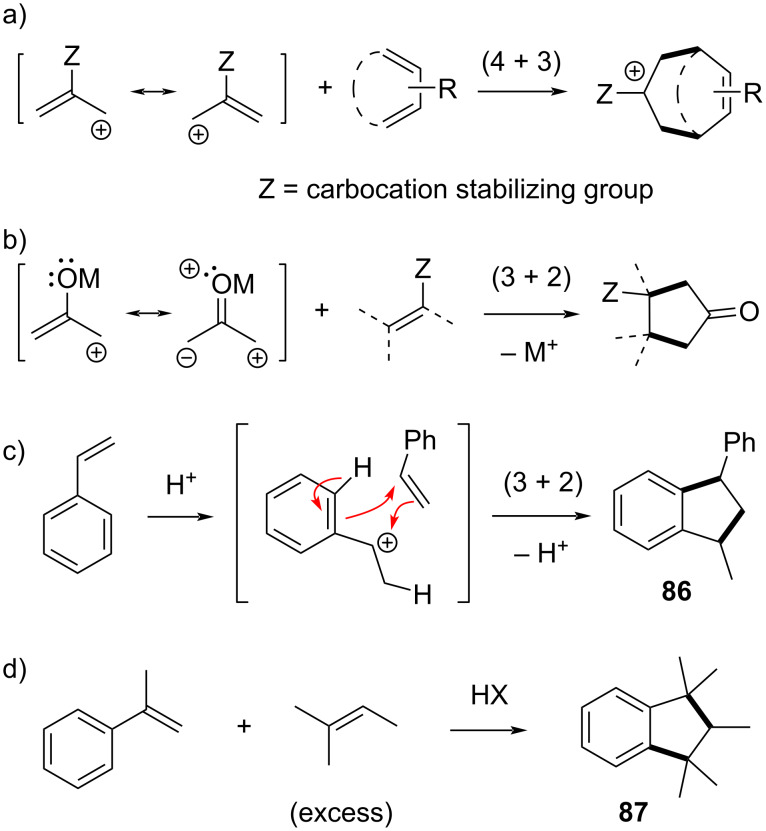
Known cycloaddition reactivity modes of allyl cations [84–100].

Allyl cations are versatile electrophiles for the allylation of various nucleophiles, but can also act as C3-π-systems in a range of cycloaddition reactions (Scheme 13) [84–88]. When combined with dienes, a swift (4 + 3) cycloaddition can happen, which is isoelectronic to a Diels–Alder reaction, and amounts to a homo-Diels–Alder reaction (Scheme 13a) [89–90]. Cycloaddition reactions of ‘naked’ unsubstituted allyl cations are usually not synthetically useful, as they lead to a mixture of products related to different competing reaction pathways. However, when suitable cation-stabilizing substituents are present, especially on the central carbon atom, very useful transformations can result for the rapid assembly of cycloheptanoid scaffolds [91]. Oxyallyl cations (when Z is an oxygen-based group) are especially favored here [92–93], and these allyl cations can also be seen as 1,3-dipoles, cross-conjugated by a carbonyl (Scheme 13b). As can be expected from this 1,3-dipolar nature, such amphiphilic allyl cations can also be used in (3 + 2) cycloadditions, though this has so far been reported far less commonly than their use in (4 + 3) cycloadditions [94].

Simple hydrocarbon allyl cations can also undergo (3 + 2) cycloadditions through a purely stepwise cation olefin cyclization-type pathway, but these generally give complex mixtures and low yields and show unpredictable substrate dependence [95]. On the other hand, simple benzyl cations can undergo more controlled (3 + 2) annulations, as is illustrated by the long-known Friedel–Crafts-type acid-catalyzed cascade reaction of styrene leading to the cyclic styrene dimer **86** (Scheme 13c) [96–97]. This (3 + 2) cycloaddition reactivity of benzyl cations can also be extended to ‘mixed’ cycloadditions [98], as is illustrated by the commercially exploited synthesis of the common pentamethylindane-derived perfumes (Scheme 13d) [99–100]. The popular perfume building block **87** is readily obtained from α-methylstyrene and an excess of amylene to suppress homodimerization, via a stepwise (3 + 2) cycloaddition of the initially generated cumyl cation across the olefin in amylene.

**Scheme 14:**
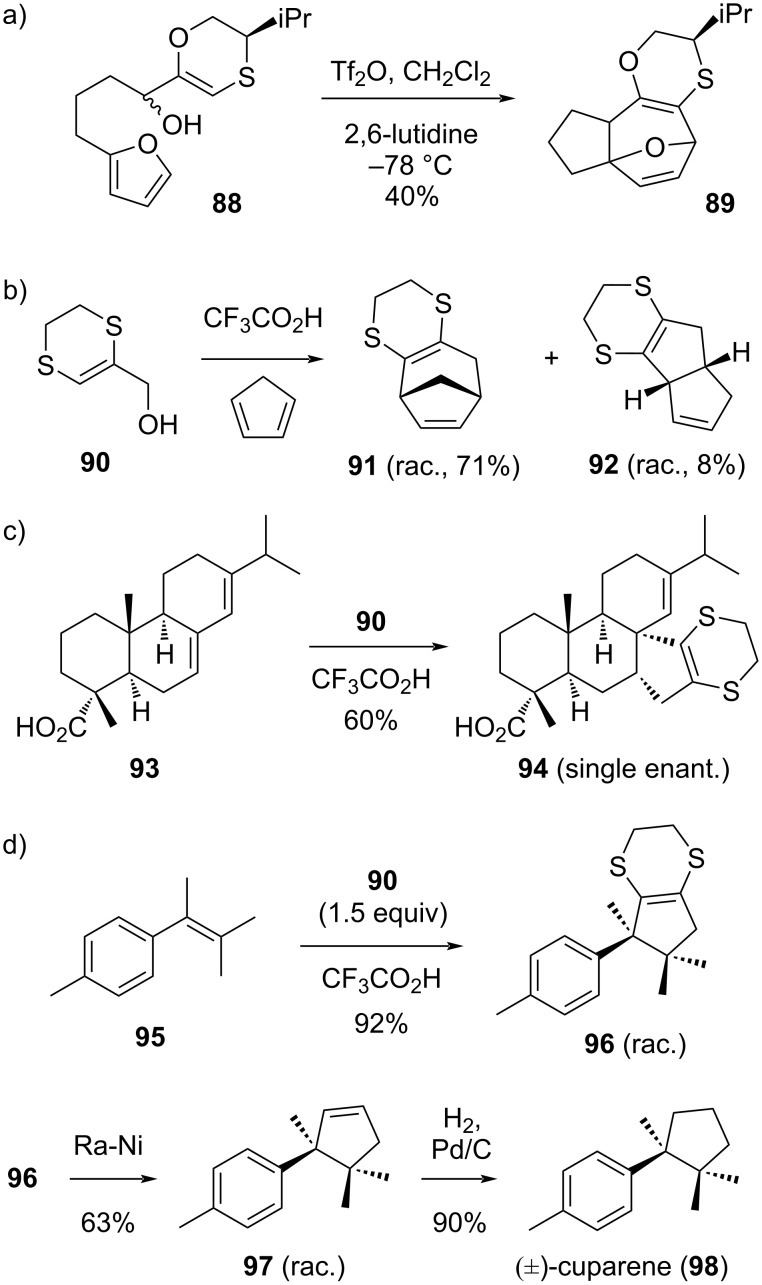
Cycloadditions of 1,4-dithiane-fused allyl cations derived from dihydrodithiin-methanol **90** [101–107].

Our group became intrigued by the potential of dihydrodithiins to act as carbocation-stabilizing groups, as these would represent a synthetic equivalent of ‘naked’ allyl cations in cycloadditions. Harmata has briefly investigated the use of oxathiin-stabilized allyl cations with some success in intramolecular (4 + 3) cycloadditions (viz **88** → **89**, Scheme 14a) [101–102]. Later, our group found that dihydrodithiinmethanol **90** acts as a very useful precursor for a range of intermolecular allyl cation-type cycloadditions (Scheme 14b–d) [103]. At first, this was investigated for its use in typical (4 + 3) cycloadditions with dienes, but mixed results were obtained (Scheme 14b). Whereas other heteroatom-stabilized allyl cations, such as oxathiins developed by Harmata, or furans developed in our own group [104–107], give exclusive (4 + 3) cycloadditions with dienes, we found that dihydrodithiinmethanol gave competitive (3 + 2) cycloadditions even with highly activated dienes such as cyclopentadiene (viz **92**). Usually, (4 + 3) cycloaddition pathways are strongly favored over (3 + 2) cycloaddition pathways, leading us to investigate the use of the 1,4-dithiane-fused allyl alcohol **90** (and its derived carbocation) in purposeful (3 + 2) cycloadditions.

In our studies of the cycloaddition reactivity of dihydrodithiinmethanol **90** [103], we found that single*-trans*-locked dienes very cleanly give the cyclopentannulated products in good yields, and with excellent stereo- and regioselectivity (Scheme 14c), as demonstrated by the reaction of abietic acid (**93**), affording **94** as a single regio- and stereoisomer. Similarly, a wide range of styrene substrates smoothly underwent the (3 + 2) cycloaddition pathway under simple acid-promoted conditions, without notable problems of styrene oligomerization or homodimerization. The method allowed a short racemic total synthesis of the sesquiterpenoid cuparene (±-**98**, Scheme 14d), wherein the synthetic challenge of the contiguous quaternary centers is tackled by the direct cycloaddition of allyl cation **90** across a tetrasubstituted olefin **95**, at the same time elaborating the cyclopentane core of this natural product. The completion of this short synthesis also nicely showcases the use of dihydrodithiinmethanol **90** as a synthetic equivalent of a simple allyl cation in (3 + 2) cycloadditions.

**Scheme 15:**
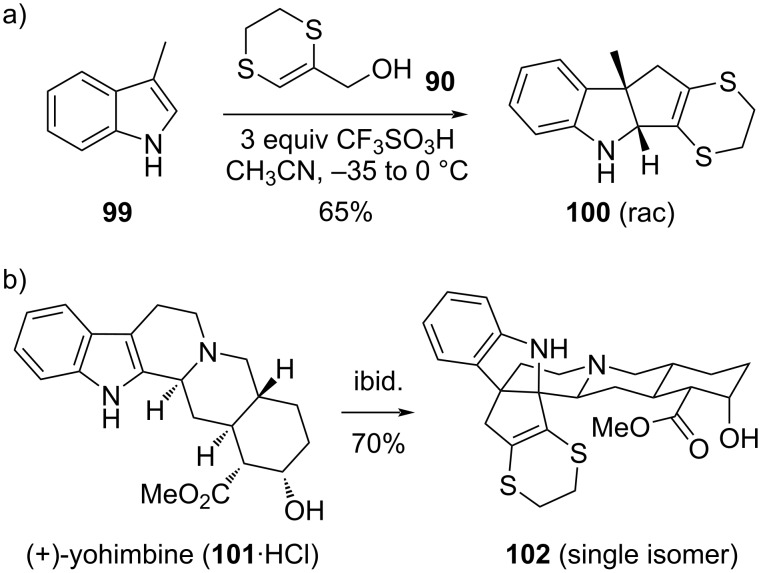
Dearomative [3 + 2] cycloadditions of unprotected indoles with 1,4-dithiane-fused allyl alcohol **90** [30].

More recently, our group was able to extend the scope of dihydrodithiin-mediated cycloadditions to indole substrates [30]. Indoles are formal styrene analogs, with very different electronic properties and reactivity profiles, and initially gave very poor results with dihydrodithiinmethanol, with incomplete conversion to complex mixtures of diverse addition products. However, we found that the reactions of allyl alcohol **90** with indoles become very reliable and quite general when a large excess of a very strong Brønsted acid is used (Scheme 15a). The use of the much stronger triflic acid actually paradoxically protects the rather sensitive indoline cycloaddition products such as the dearomatized product **100**, derived from skatole (**99**), by keeping the adduct and all intermediates in their protonated form throughout the progress of the reaction. This protonation effectively prevents any possible side reactions of the dearomatized products with electrophiles. A remarkably wide substrate scope was observed for this dearomative indole cyclopentannulation reaction, as demonstrated by the smooth ring expansion of the natural alkaloid drug yohimbine (**101**), without the need for any protecting groups (Scheme 15b).

**Scheme 16:**
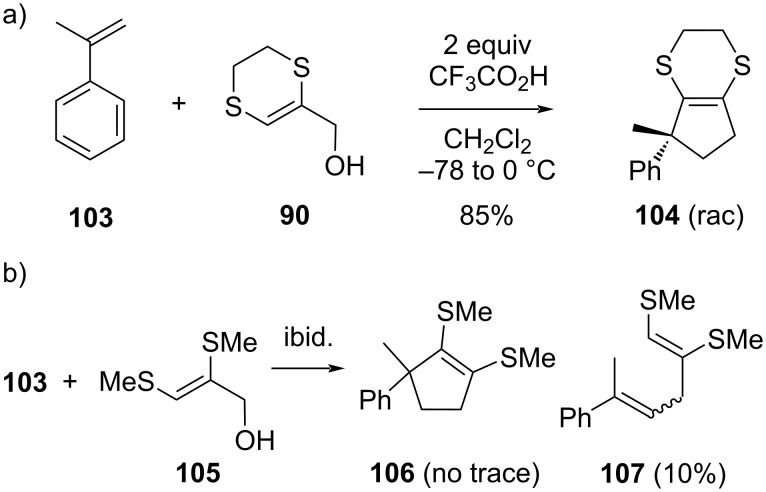
Comparison of reactivity of dithiin-fused allyl alcohols and similar non-cyclic sulfur-substituted allyl alcohols.

Our group has also investigated non-cyclic analogs of 5,6-dihydro-1,4-dithiin-2-ylmethanol (**90**), such as the dimethylthio-substituted allyl alcohol **105** (Scheme 16) [103]. Surprisingly, we have found that these allyl alcohols totally lack the otherwise observed cycloaddition reactivity (compare reactions of α-methylstyrene (**103**) in Scheme 16b and 16a). With a range of substrates that were reacted with **105**, only very low conversions of the olefins were observed (≈10%), and not even a trace of the expected cyclopentannulated products (viz **106**) was formed. In part, this was found to be due to decomposition (and self-condensation reactions) of the dimethylthio-substituted allyl alcohol **105**. However, also the minor adducts that were formed with the olefins were shown to be mixtures of exclusively non-cyclic allylation products of the starting material (viz **107**). These observations are more in line with the reactivity observed for normal allyl cations (where oligomerizations to linear dimers are also the main reaction pathways). The discrepancy between the reactivity of allyl alcohols **99** and **105** can perhaps again be related to the special ‘pseudoaromatic’ character of the dihydrodithiin ring which has a tendency to remain intact as a conjugated bonding array (cf. chapter 2, also compare to Scheme 13c and 13d).

**Scheme 17:**
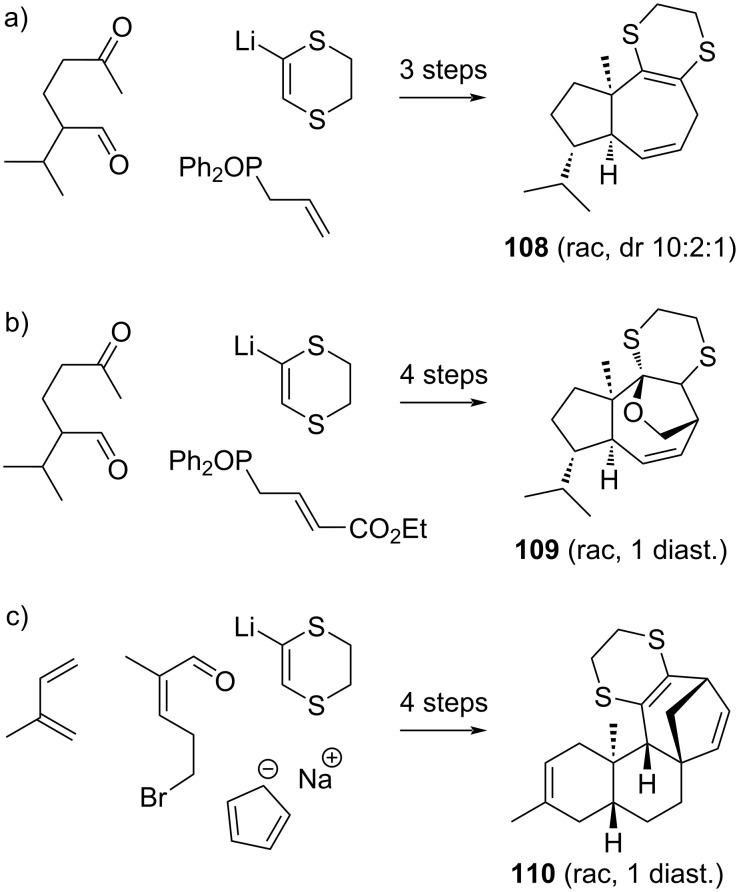
Applications of dihydrodithiins in the rapid assembly of polycyclic terpenoid scaffolds [108–109].

The scope of the dihydrodithiin-based allyl cation cycloadditions can be appreciated by the relative ease with which it allowed the assembly of complex terpenoid frameworks. Examples from our research group include various daucanoid and kauranoid terpene scaffolds such as **108**, **109**, and **110**, which can be assembled from dihydrodithiin **3** as a building block in just a few synthetic operations with good to excellent control of the relative stereochemistry (Scheme 17) [108–109].

### 6 Synthetic equivalents of vinyl carbenes in (2 + 1) cycloadditions: Au(I)-catalyzed generation of 1,4-dithiane-fused vinylcarbene species

**Scheme 18:**
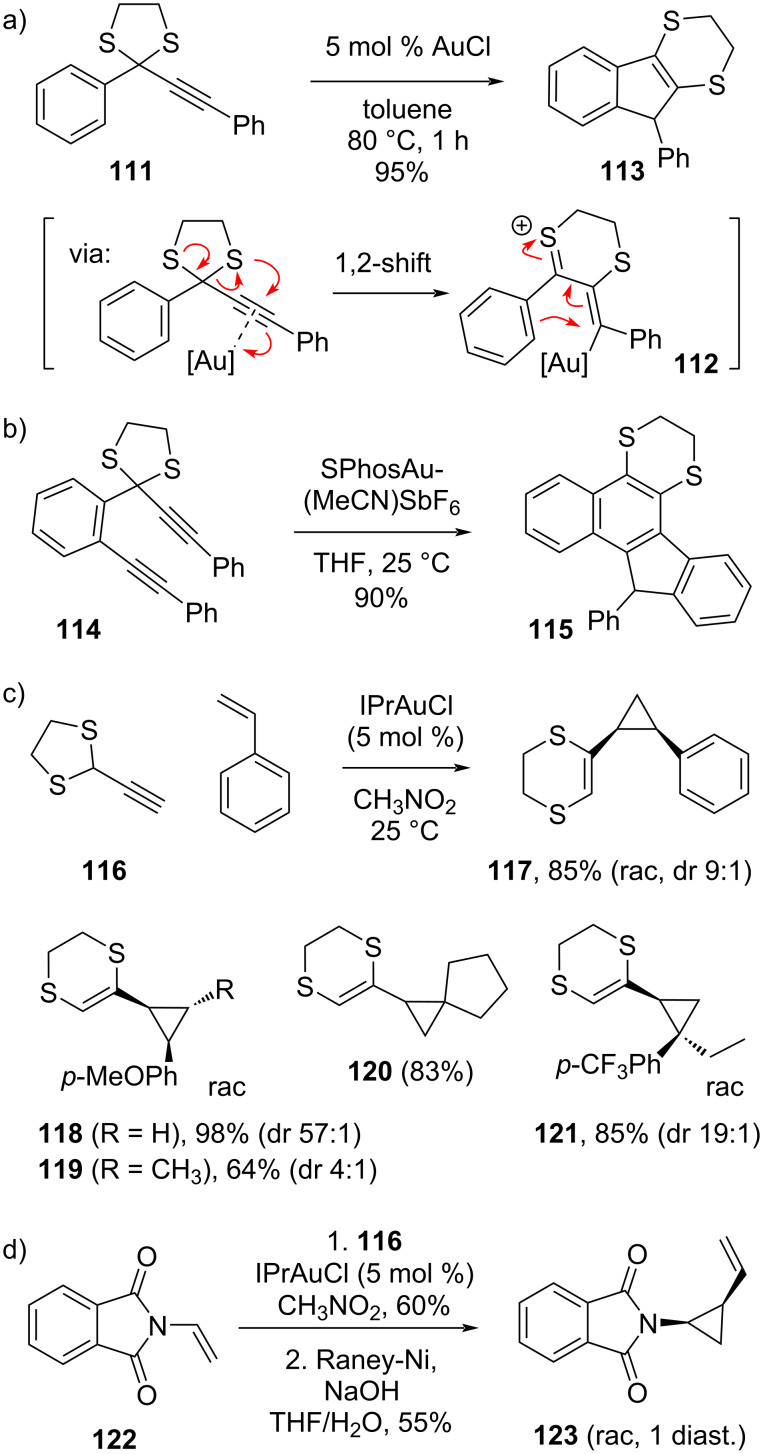
Dihydrodithiin-mediated allyl cation and vinyl carbene cycloadditions via a gold(I)-catalyzed 1,2-sulfur-migration.

In 2007, Wang and co-workers reported a gold-catalyzed Parham-type 1,2-sulfur migration to generate in situ a dithiinyl cation-type reagent from an alkyne-substituted 1,3-dithiolane (Scheme 18a, also compare to Scheme 4) [110]. The rearranged vinylthionium cation **112** was not isolated, but was here postulated to explain the formation of the Nazarov-type reaction product **113** that was isolated in excellent yield, and which is likely formed directly from the allyl cation/vinylthionium-type intermediate **112**. In 2016, Liu and co-workers extended this interesting reactivity mode to a cascade polyyne cyclization of 1,3-dithiolane **114** to 1,4-dithiane **115** (Scheme 18b) [111]. These authors also investigated mechanistic probes that indicated the carbocationic nature of the involved intermediate species.

**Scheme 19:**
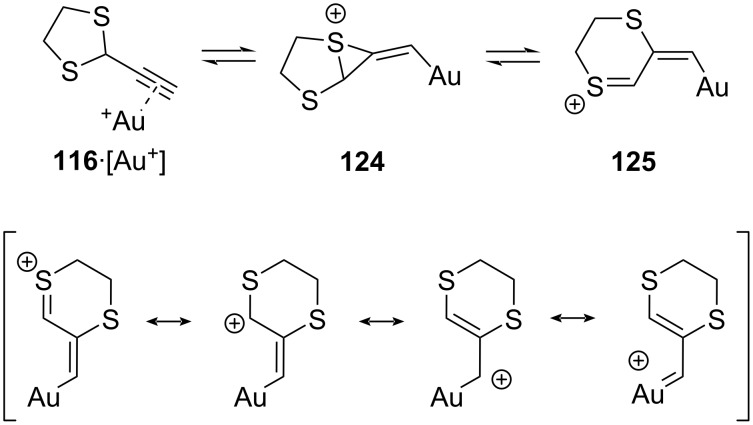
Activation mode of ethynyldithiolanes towards gold-coordinated 1,4-dithiane-fused allyl cation and vinyl carbenoid reagents.

Spurred by our results in (3 + 2) cycloadditions of 1,4-dithiane-fused allyl cations (vide supra) [103], our group recently investigated the use of Wang’s soft activation mode of 1,4-dithiane-fused allyl cations for its use in intermolecular cycloadditions, as the putative gold(I)-coordinated intermediates like **112** are indeed quite similar to those expected to arise from dihydrodithiin alcohol **90** (see Scheme 19) [112]. However, our results with the simple 2-ethynyl-1,3-dithiolane (**116**) immediately showed that the expected allyl cation (3 + 2) cycloaddition reactivity is not operating under gold(I) catalysis, but instead it behaved as a reliable and quite stereoselective vinylgold carbenoid species, affording exclusively cyclopropanation products with a wide range of olefin substrates (Scheme 18c). The carbene-type nature can be appreciated in the resonance structures of vinylthionium species **125**, wherein the positive charge is relegated to the gold center (Scheme 19). The reactions of dithiolane **116** also work with unactivated (viz **120**) and heteroatom-substituted olefins (viz **122**) and were found to generally favor formation of the *cis*-vinylcyclopropanes (Scheme 18c and 18d). Desulfurization of the adducts again yields the product of a formal cyclopropanation with a ‘naked’ vinyl carbene species, as demonstrated by the stereoselective synthesis of the protected *cis*-2-vinylcyclopropan-1-amine **123** (Scheme 18d).

### 7 Downstream chemistry and further applications: deprotection, cleavage or further functionalization of 1,4-dithianes

**Scheme 20:**
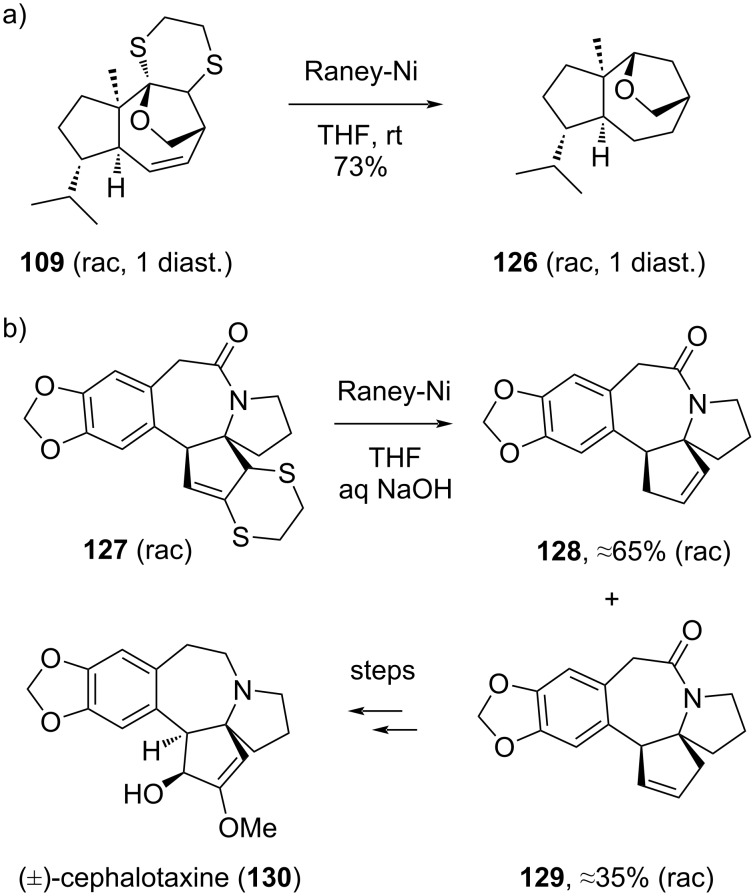
Desulfurization problems.

In organic synthesis, the deprotection of 1,3-dithianes has a reputation of being a troublesome reaction. In the chemical literature, there are probably well over a hundred distinct procedures to be found for the deprotection of dithioketals [113]. This is likely because none of them is really generally applicable, and synthetic chemists have often found themselves in a place where they were in need to find an alternative procedure for their particular substrate. Nonetheless, the distinct reactivity of the sulfur atoms does allow many mild and chemoselective manipulations. For 1,4-dithianes, the literature for selective functional group transformations and deprotections is obviously less extensive than for 1,3-dithianes, but the various reports that can be found also point towards the fact that no standard set of conditions will suffice to achieve the desired transformations [42,58–59]. This is certainly our own experience in our lab. The optimal reaction times, additives, pH, solvents, and reagents (including reagent grades) can be surprisingly substrate dependent. Nevertheless, given some experimentation, the desired chemoselective transformation can be achieved in almost all cases, including for sensitive substrates such as yohimbine-derived compound **102** [30]. A common problem is the concomitant hydrogenation of alkenes, which can be hard to avoid, as seen in the hydrodesulfurization of the daucanoid scaffold **109** (Scheme 20a) [108]. For the vinylcyclopropane desulfurizations (cf Scheme 18d) [112], this overreduction phenomenon was particularly troublesome, and reactions had to be very closely monitored, and stopped well before complete consumption of the starting material. This was required to avoid undesired hydrogenation of both the vinyl and the cyclopropane moieties, which both proved sensitive to the action of Raney nickel.

A particularly troublesome episode that demonstrates the problems one can encounter in dithiane desulfurizations, was encountered in our labs during our (formal) total synthesis of (±)-cephalotaxine (**130**, Scheme 20b) [112]. Here, the desired desulfurization of a 1,4-dithiane could not be achieved without concomitant migration of an alkene double bond (viz **127** → **128**), making the final steps to complete the synthesis quite cumbersome, as at best mixtures of the desired and undesired positional alkene isomer could be obtained.

We believe an outstanding challenge in the field of chemical synthesis is to find mild and catalytic deprotection chemistries for dithiane-type systems, be they reductive, hydrolytic or oxidative. In particular, a more controlled alternative for the often troublesome, but nevertheless highly chemoselective hydrodesulfurization with Raney nickel would be a highly valuable addition to the organic synthesis tool box. Few good or general alternatives for this method seem to exist [114], and for very sensitive substrates, control of the reactivity of Raney nickel (which always has to be employed in excess and in a heterogenous system), can be quite time consuming.

**Scheme 21:**
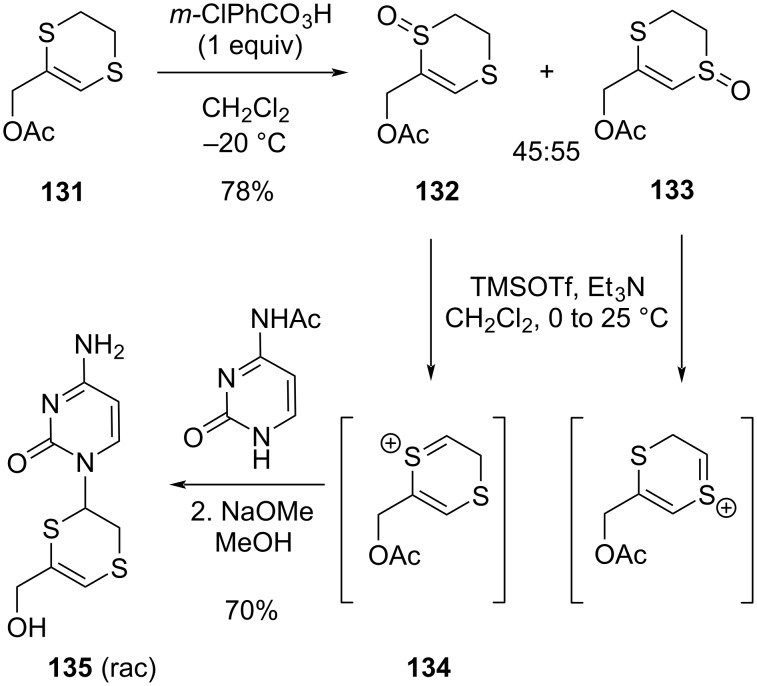
oxidative decoration strategies for 1,4-dithiane scaffolds.

Finally, the 1,4-dithiane or -dithiin ring system need not necessarily be considered as a temporary tethering of protecting group, but can also become an integral part of synthetic targets, or can again serve as a handle for further derivatization. An obvious choice here is to oxidize the sulfur atoms to give (vinyl)sulfones, which can be used in a range of further bond formations, in particular Diels–Alder reactions (cf. chapter 3, Scheme 8). In this context, 1,4-dithianes can thus also be considered as synthetic equivalents of cyclohexanes. Oxidative decoration of the carbon atoms in the dithiane ring can also be achieved via Pummerer-type chemistry, as illustrated by Pallumbo’s racemic synthesis of the dihydrodithiin-based nucleoside analog **135** via an oxidation of one of the ring sulfur atoms in a dihydrodithiin building block (Scheme 21) [115]. Controlling the regioselectivity of the monooxidation is often not trivial, but the sulfoxides **132** and **133** can be separated and allow access to various derivatives through addition of a suitable nucleophile to the putative sulfonium-type dithiin intermediates such as **134**. It is noteworthy that these Pummerer rearrangement-generated sulfonium intermediates do not give the expected fully unsaturated dithiin rings, but can be captured by nucleophiles before elimination of a proton happens.

## Conclusion

In this topical review, we have aimed to show the reader the synthetic potential of 1,4-dithiane-type building blocks. We have focused in particular on applications in which they allow the rapid and controlled assembly of otherwise hard to achieve molecular frameworks. We believe there is still great untapped potential in this area of research, and expect the utility of these building blocks to grow in the coming years. In particular, the recently established options to elaborate and functionalize 1,4-dithianes using mild transition-metal-catalyzed couplings, and their use in cycloaddition reactions, should open up many more possibilities. Moreover, as the synthetic access to these sulfur-based heterocycles is now more straightforward, the importance of 1,4-dithianes and related heterocycles in new synthetic methodologies can be expected. An area where these C2-synthons have so far been less extensively explored, is in asymmetric synthesis, although this should be quite feasible. We hope this review can help inspire such future developments.
